# Label-Free Electrochemical Cell-Based Biosensor for Toxicity Assay of Water-Soluble Form of Phosphatidylcholine

**DOI:** 10.3390/biomedicines13040996

**Published:** 2025-04-20

**Authors:** Veronica V. Pronina, Lyubov V. Kostryukova, Sergey V. Ivanov, Elena G. Tichonova, Alexander I. Archakov, Victoria V. Shumyantseva

**Affiliations:** 1 Institute of Biomedical Chemistry, Pogodinskaya Street, 10, Moscow 119121, Russia; veronicapunch@mail.ru (V.V.P.); kostryukova87@gmail.com (L.V.K.); ivanov-sv-tver@mail.ru (S.V.I.); elena.tikhonova_@mail.ru (E.G.T.); alexander.archakov@ibmc.msk.ru (A.I.A.); 2 Faculty of Biomedicine, Pirogov Russian National Research Medical University, Moscow 117997, Russia

**Keywords:** cell-based sensor, electrochemical evaluation, HepG2 cells, Wi-38 cells, phosphatidylcholine, cytotoxicity

## Abstract

**Background/Objectives:** Our study brings a new method to properly evaluating drug efficacy at the non-invasive in vitro level. **Methods:** In this work, the electrochemical mediator-free and reagent-free analysis of cell lines based on the registration of electrochemical profiles of membrane proteins was developed. We studied the specificity of cell lines Wi-38 and HepG2 and the toxic effects of drugs on cell-on-electrode systems. **Results:** A linear dependence of the peak current on the concentration of cells applied to the electrode in the range from 1 × 10^5^ to 6 × 10^5^ cells/electrode was registered (R^2^ 0.932 for Wi-38 and R^2^ 0.912 for HepG2). The water-soluble form of phosphatidylcholine (wPC) nanoparticles recommended for atherosclerosis treatment and prevention of cardiovascular diseases did not show a toxic effect on the human fibroblast cells, Wi-38, or the human hepatocellular carcinoma cells, HepG2, at sufficiently high concentrations (such as 0.1–1 mg/mL). The antitumor drug doxorubicin, at concentrations of 3 and 10 μg/mL, showed a pronounced toxic effect on the tested cell lines, where the percentage of living cells was 50–55%. **Conclusions:** A comparative analysis of the cytotoxicity of wPC (0.1–1 mg/mL) and doxorubicin (3–10 μg/mL) on the cell lines Wi-38 and HepG2 using the MTT test and electrochemical approach for the registration of cells showed their clear adequacy.

## 1. Introduction

Aging processes and increasing the active human lifespan are among the main tasks of gerontology and humanity as a whole [1,2,3]. Every year, a large number of people die from various diseases (cardiovascular, oncological, neurodegenerative, etc.) all over the world [4]. It is believed that the occurrence of various diseases and their aggressive development occurs during the aging process. The aging process can be associated with many signs, such as genomic instability, loss of proteostasis, disruption of nutrient perception systems, mitochondrial dysfunction, cellular aging, decreased self-renewal capacity [5], and an increase in cardiovascular diseases associated with the manifestation of atherosclerosis [6,7,8,9].

Liver cells have a high regenerative capacity. However, during the aging process, after damage the cells activate compensatory mechanisms, which, when hyperstimulated, can lead to a decrease in regenerative capacity and the development of pathologies [5]. Cellular aging is one of the causes of aging, in which an irreversible stop of the cell cycle of proliferating cells occurs. In liver fibrosis, aging stellate cells were found, in which there is a decrease in collagen production and proliferation. The occurrence of fibrosis can be a consequence of chronic liver diseases of various etiologies (toxic damage; viral infections; and autoimmune, metabolic, and genetic disorders) [10,11]. To reduce the risk of fibrosis and increase lifespan, timely treatment of concomitant diseases, including liver diseases, is necessary.

Phospholipids are key elements in mammalian cells. For example, phosphatidylcholine (PC) is the main component of cell membranes and subcellular organelles [12]. The similarity of plant phospholipids to phospholipids of mammalian cell membranes allows them to be included in these structures for the prevention of a number of pathological processes [13,14]. When taking phospholipids of plant origin (for example, soy phospholipids), a decrease in lipid levels is observed, the level of cholesterol and triglycerides in the blood is controlled, membrane function is stabilized, and liver health is supported [13]. When developing drugs, one of the important criteria for their release on the pharmaceutical market is their biocompatibility, biosafety, and low toxicity [15]. The pharmacological properties of drugs are studied on various models, such as enzymes, DNA, antibodies, and cells [12,13,14] in in vivo and in vitro systems using modern physicochemical and biochemical methods [16,17,18,19]. One of the developing areas in drug research is their electrochemical analysis [20,21]. Due to their high accuracy, sensitivity, low cost, rapidity, flexibility of analysis, and non-invasive format, electrochemical methods have shown promise for their use in in vitro experiments on various biological objects, including cell lines [22,23,24]. The use of electrochemical biosensors allows one to identify chemical compounds as potential drugs and investigate the biological mechanism of drugs [23,24]. Electrochemical sensors allow the registration of trace amounts of analyzed compounds with non-destructive and in situ detection approaches [25,26,27]. Earlier, we studied electrochemical DNA biosensors and cytochromes, P450-biosensors, and interactions of these sensor systems with drugs [28,29,30,31]. Cell-based biosensors are promising models for the investigation of drug toxicity.

In electrochemical studies, prokaryotic and eukaryotic cells are used as sensitive elements for both the detection and registration of the cells themselves and for the assessment of the influence and impact of various toxic compounds or medicinal preparations on cells by measuring the transformation of the electrochemical response of the bio object [32]. The aim of our work was to study the influence of the water-soluble form of phosphatidylcholine (wPC) nanoparticles on normal human fibroblast cells, Wi-38, and on liver cells of human hepatocellular carcinoma, HepG2. The novelty of our investigation lies in the in situ/process monitoring of drug intervention on cell lines using electrochemical monitoring. The developed electrochemical assay showed cell-surface proteins’ profiles and the dose-dependencies of wPC on these cell lines.

## 2. Materials and Methods

### 2.1. Materials

In experiments, we used the water-soluble pharmaceutical form of PL nanoparticles as ultra-small phospholipid micelles from soybean phosphatidylcholine, with an average size of 30 nm (wPC), which contained 0.5 g of phosphatidylcholine and 2 g of D-maltose [33,34,35] as a stock solution in 100 mL of 0.1 M potassium phosphate, pH 7.4, containing 0.05 M NaCl (PBS). A water-soluble form of phosphatidylcholine (wPC) is a lyophilized powder that produces an ultrafine emulsion upon rehydration. Maltose monohydrate from Sigma-Aldrich (St. Louis, MO, USA) was used as a control, acting as a cryoprotective agent to preserve the properties of NPs after lyophilic drying during rehydration. The cytostatic doxorubicin hydrochloride (g/h) (JSC Omutninskaya Scientific Pilot-Industrial Base, Omutninskaya, Russia) was chosen as a well-known cytotoxic agent [16].

The following reagents were used: single-walled carbon nanotubes (SWCNTs; diameter 1.6 ± 0.4 nm, length > 5 µM, surface area 1000 m^2^/g), TUBALL™ BATT H_2_O, with water dispersion of 0.4%, stabilized by carboxymethylcellulose, were obtained from OCSIAL Ltd. (https://ocsial.com, accessed on 17 January 2025, Novosibirsk, Russia); potassium phosphate monobasic (≥99%) was purchased from Sigma-Aldrich. Sodium chloride (99.5%) was purchased from Acros Organics (Carlsbad, CA, USA). All aqueous solutions were prepared using Milli-Q water (18.2 MΩcm) purified with a Milli-Q water purification system by Millipore.

Electrochemical experiments were performed in a supporting electrolyte 0.1 M potassium phosphate buffer with 50 mM NaCl (PBS, pH 7.4).

### 2.2. Cell Lines

In the current study, cell lines of human fibroblasts, Wi-38, and human hepatocellular carcinoma of the liver, HepG2, obtained from the American Type Culture Collection (ATCC) and stored in the IBMC cell culture collection, were used.

The cells were incubated in Dulbecco’s modified Eagle’s medium (DMEM) (PanEco, Moscow, Russia) with 2 mM L-glutamine and 10% FBS (fetal bovine serum) (Capricorne, Ebsdorfergrund, Germany) added, in 25 cm^2^ and 75 cm^2^ culture flasks (Biologyx, Jinan, Shandong, China) at 37 °C, in a humid atmosphere with 5% CO_2_ (CO_2_ incubator, Sanyo, Moriguchi, Osaka, Japan). Versene solution and a 0.25% trypsin solution were used during the passage to detach the cells, adding them to the flask for 2–3 min. The cells were used after 3–18 passages and freezing.

### 2.3. Study of Cytotoxicity of Water-Soluble Form of Phosphatidylcholine (wPC) by MTT Test In Vitro

The cytotoxicity of the water-soluble form of phosphatidylcholine (wPC) was assessed in vitro using the MTT test. Wi-38 and HepG2 cells (10^5^ cells per well) were seeded in sterile 96-well culture plates (Biologyx, Jinan, Shandong, China) and incubated at 37 °C in 5% CO_2_ using a CO_2_ incubator (Sanyo) for 24–26 h. Then, the test samples were added at wPC concentrations of 1 mg/mL and 0.1 mg/mL. Maltose, a component of the water-soluble form, was used as a control and added at concentrations of 4 mg/mL and 0.4 mg/mL. The cells with the test substances were incubated for 24 h. After that, 50 μL of MTT reagent (1 mg/mL) (Mumbai, Mumbai, Maharashtra, India) was added and incubated at 37 °C for 3 h, after which they were removed, and 100 μL of DMSO (PanEco, Moscow, Russia) was added. The cells were covered with foil and shaken on an orbital shaker for 15 min. Absorbance was recorded at 570 nm using a Multiscan FC (ThermoSpectronic, Waltham, MA, USA) and normalized to the untreated control (no substances).

### 2.4. Electrochemical Study of wPC Influence on Cell Lines

Electrochemical measurements were carried out using Autolab 302N potentiostat/galvanostat (Metrohm Autolab, Utrecht, The Netherlands) equipped with the NOVA software (version 2.0). Commercially available screen-printed electrodes (SPEs) were obtained from ColorElectronics, Moscow, Russia (http://www.colorel.ru, accessed on 17 January 2025). The SPEs contained graphite working electrodes (geometric area 0.0314 cm^2^), auxiliary electrodes, and silver/silver chloride reference electrodes (Ag/AgCl). All potentials were referred to the Ag/AgCl reference electrode. Then, 2 μL of diluted SWCNT TUBALL™ BATT H_2_O dispersion (0.75 ± 0.05 mg/mL) was applied to the surface of the electrodes. Electrodes SPE/CNT stayed at room temperature until completely dried [28,29].

Wi-38 and HepG2 cells were inoculated (5 × 10^5^ cells per well) into 12-well plates (Biologyx) and cultured at 37 °C in an atmosphere with a relative humidity of 95% and 5% CO_2_ for 24 h. Cells were incubated with wPC (1 mg/mL, 0.1 mg/mL), maltose monohydrate (4 mg/mL and 0.4 mg/mL) or doxorubicin (3 µg/mL, 10 µg/mL) 24 h, at 37 °C in the CO_2_ incubator. After the incubation, the medium with the introduced samples was removed. The cells were washed three times with Versene solution and detached as described above. The cell suspension was washed twice with phosphate-buffered saline (PBS; PanEco) and precipitated in an Elme (Riga, Latvia) centrifuge at 1000× *g* for 4 min, followed by removal of the supernatant. The cell pellets were resuspended with potassium phosphate buffer to equal cell concentrations in µL. Cell counting was performed using the ViCell XR analyzer (Beckman Coulter, Brea, CA, USA).

The square wave voltammetry (SWV) was recorded for the registration of cells and cells treated with water-soluble form of phosphatidylcholine (wPC), maltose, or doxorubicin. The following optimized SWV parameters were used: 0.0 to +1.0 V, step 5 mV, modulation amplitude 0.02 V, and frequency 10 Hz. For electrochemical measurements, 2 μL of the test solution was applied onto the surface of the modified electrode and incubated at room temperature for 25 min. A horizontal measurement regimen was used for all electrochemical experiments under aerobic conditions at room temperature. A 60 µL drop of PBS was placed onto the SPEs to cover the surface of all three electrodes. Incubation was carried out on the electrode in a drop of 60 µL for 5 min before the start of the experiment. To assess the reproducibility of the results for each concentration, at least 3 electrodes were used, and the standard deviation was calculated.

The cytotoxicity of the samples obtained by the electrochemical method was calculated using the following formula [27,36]:

(1) Cytotoxity(% )=(I(cells−drug) /I(cells))×100%

where cytotoxicity, (%)—electrochemical coefficient of cytotoxic effect, I(cells – drug)—peak current of cell after interaction with compound, I(cells)—peak current of cell before interaction with compound (used as control).

### 2.5. Statistical Analysis

Student’s test was used to assess the significance of differences in the electrochemically measured parameters over three measurements. Differences were considered statistically significant at *p* ≤ 0.05. In the figures, the data are presented as the mean value ± standard error relative to the mean value.

## 3. Results

Aging is a complicated biological process, which reflects a systemic response to a gradual accumulation of cellular and tissue damage and dysregulation of defense systems [2,37,38]. The use of drugs and geroprotectors involves assessing their toxic effects on both the liver and the body as a whole. Nanoparticles (NPs) of various chemical natures and compositions are currently widely used in drug delivery, diagnostics, cosmetology, gerontology, and a number of other areas [37]. Because of the fact that a large number of drugs are being developed based on nanoparticles, and many studies are currently being conducted in this area, the question of the safety of using NPs in drug development arises. Thus, it is important to assess the safety of such drugs and the biocompatibility of the NPs used [38]. Due to their size, nanoparticles are able to overcome various biological barriers inside the body, in particular the blood–brain barrier. In addition, the nanosize of the particles in the drug provides its access to the cell and its various compartments, including the nucleus [39]. Interactions with cells and the toxicity of NPs largely depend on their composition.

To assess the toxicity of the water-soluble pharmaceutical form of PL nanoparticles as ultra-small phospholipid micelles from soybean phosphatidylcholine with an average size of 30 nm (wPC) in in vitro studies, the normal human fibroblast line Wi-38 and the human hepatocellular carcinoma tumor line HepG2 were selected as model cell lines. We used these cell lines as liver-on-electrode models for the assessment of the concentration-dependent toxic effect of wPC and doxorubicin by means of a standard method (MTT test) and electrochemical analysis of the Wi-38 and HepG2 cell lines, which differ significantly in morphology and size.

In our investigation, we used two methods for comparing the toxicity assessment of the water-soluble form of phosphatidylcholine: (i) 3-(4,5-dimethylthiazol-2-yl)-2,5-diphenyltetrazolium bromide (MTT) assay (MTT test) and (ii) electrochemical bioassay based on monitoring the response of cell lines to interaction with the drug doxorubicin or wPC.

### 3.1. Cytotoxicity of Water-Soluble Form of Phosphatidylcholine wPC by MTT Test

**Figure 1:**
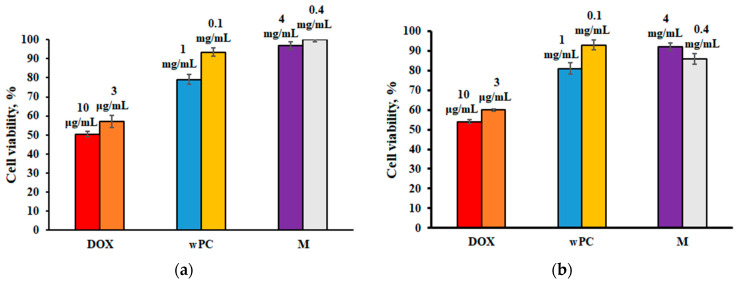
Cell viability of (**a**) Wi-38 cells and (**b**) HepG2 cells after 24 h incubation with doxorubicin (DOX), water-soluble form of phosphatidylcholine (wPC), and maltose monohydrate (M) at different concentrations. Data are mean ± standard deviation (n It is correct  = 6).

The water-soluble form of phosphatidylcholine wPC is a powder that forms an ultrafine emulsion with a particle size of less than 30–50 nm upon rehydration [34]. The studied water-soluble form of phosphatidylcholine, cryoprotective agent (maltose), and doxorubicin (cytotoxic agent) were incubated with cells and analyzed using the MTT test (Figure 1).

A study of the cytotoxic effect of the water-soluble form of phosphatidylcholine (wPC) by the MTT test after 24 h of incubation with normal human fibroblast cells Wi-38 (Figure 1a) showed a slight decrease in the percentage of viable cells at a concentration of 1 mg/mL wPC (79.13 ± 2.60% cell viability), while incubation of Wi-38 cells with wPC at a concentration of 0.1 mg/mL wPC showed almost complete cell survival (93.34 ± 2.2% cell viability). A study of the cytotoxicity of the control substance, which is the cryoprotector component of wPC, maltose (4-O-α-D-glucopyranosyl-D-glucose), demonstrated the absence of pronounced toxic manifestations on Wi-38 cells when incubated with similar concentrations in wPC (4 mg/mL and 0.4 mg/mL by maltose). In contrast, a study of the cytotoxicity of a well-known toxic agent, the antitumor drug doxorubicin hydrochloride, at concentrations of 3 and 10 μg/mL showed a pronounced toxic effect on cells, where the percentage of living cells was 50–55%. This study confirms the non-selectivity of the cytotoxic effect of doxorubicin as an anticancer drug, leading to the adverse side effects on cells and other biocomponents, including DNA, as we have shown earlier [40,41].

The cytotoxicity of wPC after 24 h of incubation with the human hepatocellular carcinoma cell line (HepG2) also showed an insignificant decrease in the percentage of viable cells at a concentration of 1 mg/mL wPC (81.0 ± 2.85% cell viability) (Figure 1b). At a concentration of 0.1 mg/mL wPC, cell viability was almost 100%. It was also shown that incubation of HepG2 cells with the cryoprotective agent maltose did not lead to a significant decrease in viable cells at the studied concentrations of 4 mg/mL and 0.4 mg/mL. A study of the cytotoxicity of doxorubicin on this cell line showed almost 50% death of tumor cells, while no significant difference was observed between the studied concentrations (3 μg/mL and 10 μg/mL). Thus, MTT cytotoxicity studies of wPC as nanoparticles exhibited no significant effect on cell viability in the selected cell lines as the in vitro model.

### 3.2. Electrochemical Analysis of Cytotoxicity of Water-Soluble Form of Phosphatidylcholine (wPC)

The development of new drugs and different dosage forms demands new analytical techniques for toxicological evaluation. Cell-on-electrode assay as an in vitro cell model possesses obvious advantages, such as the 3Rs principles (Reduction in analysis duration, Replacement of chip, and Refinement of effect) [42,43].

The application of electrochemical biosensors with cells, enzymes, virus particles, or DNA immobilized on the electrode allows for detecting these biological objects with high sensitivity [44,45]. In recent years, there has been growing interest in the development of electrochemical biosensors for the analysis of cell lines (cell-based biosensors) [46,47]. Electrochemical methods are promising for assessing the cytotoxicity of drugs due to the high sensitivity of the analysis and the ability to select a rational modification to an appropriate cell line. Applying electrochemical methods allows registering changes in the electrochemical parameters of living systems (cells) before and after exposure to drugs due to the ability of biological molecules to electro-oxidize/reduce on the electrode [29,36,48,49,50]. A number of studies have used cell-based biosensors to assess the toxicity of various compounds [36,47].

In this study, the analysis of the cell lines Wi-38 and HepG2 was carried out using the square-wave voltammetry (SWV) method. Screen-printed graphite electrodes (SPEs) were modified with single-wall carbon nanotubes (SPEs/CNTs). Modification of electrodes with carbon nanomaterials leads to increased sensitivity and improved analytical characteristics of sensor elements [51,52]. Detection limits for cell registration were found depending on the cell line.

**Figure 2:**
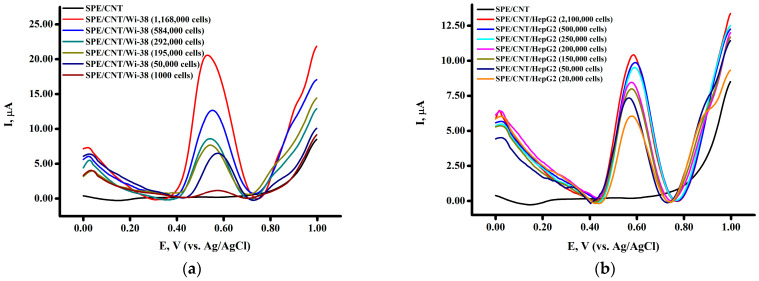

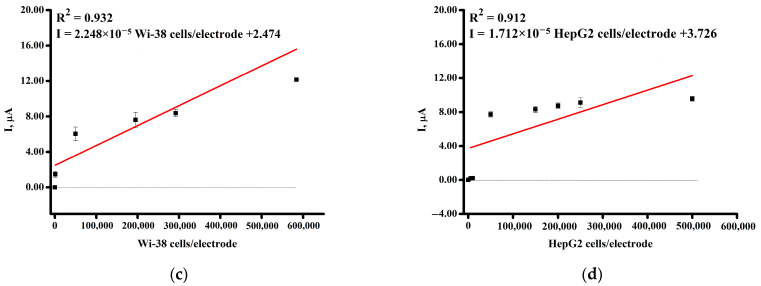
Square-wave voltammograms (SWVs) of (**a**) Wi-38 and (**b**) HepG2 cells and calibration graphs of (**c**) Wi-38 and (**d**) HepG2 cells’ dependence of peak current intensity on cell concentration. The study was carried out with SWV in the potential range from 0 to +1.05 V. Peak intensities were recorded according to the number of cells on the electrode (cells/electrode).

The linear dependence for Wi-38 or HepG2 and peak current was in the range from 1 × 10^5^ to 6 × 10^5^ cells/electrode (Figure 2c,d). The nature of the electrochemical signal of cell lines may reflect the processes of electrochemical oxidation of outer membrane proteins of cell lines [36].

The square-wave voltammograms of Wi-38 human fibroblast cells in an electrolyte buffer corresponding to physiological media (0.1 M potassium phosphate buffer with 50 mM NaCl, pH 7.4) in the potential range (0–(+)1) V revealed one intensive and clear peak at E = +(0.546 ± 0.018) V (Figure 2a). HepG2 cells (Figure 2b) were also well detected in the range of 5 × 10^4^–5 × 10^5^ cells/electrode at a potential of E = +0.577 ± 0.013 V. The limit of detection (LOD) and the limit of quantification (LOQ) were calculated from the calibration curves as kSD/b (k = 3 for LOD, k = 10 for LOQ), b = slope of the calibration curve, SD = standard deviation [53,54]. As a result, it was shown that for Wi-38 cells, LOD and LOQ were 53,809 cells/electrode and 179,365 cells/electrode, respectively. For HepG2, these parameters were 89,150 cells/electrode and 270,152 cells/electrode, respectively. However, it should be noted that when applying the HepG2 cell suspension to the electrode in higher concentrations, problems arose due to its more viscous state, which is possibly associated with the morphology of the cells.

### 3.3. Electrochemical Analysis of Water-Soluble Form of Phosphatidylcholine (wPC) and Doxorubicin on Cells

The effect of the water-soluble form of phosphatidylcholine (wPC) was studied after incubation with cells for 24 h at 1 mg/mL and 0.1 mg/mL concentrations of wPC. The cryoprotector maltose monohydrate, which is part of wPC, was analyzed at the corresponding concentrations (4 mg/mL and 0.4 mg/mL). Doxorubicin hydrochloride was used as a well-known cytotoxic agent.

**Figure 3:**
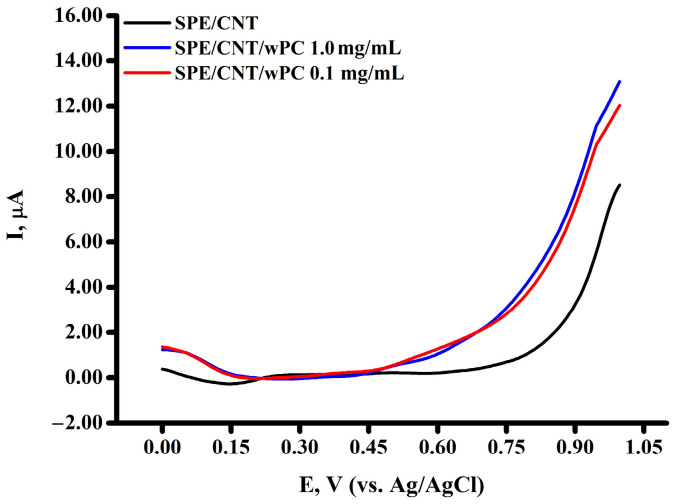
Square-wave voltammograms of SPE/CNT (−), and SPE/CNT with wPC at concentrations: 1.0 mg/mL (−); 0.1 mg/mL (−). *p* ≤ 0.05.

The square-wave voltammograms of wPC did not display any signals in the range of potentials for cell lines (Figure 3).

**Figure 4:**
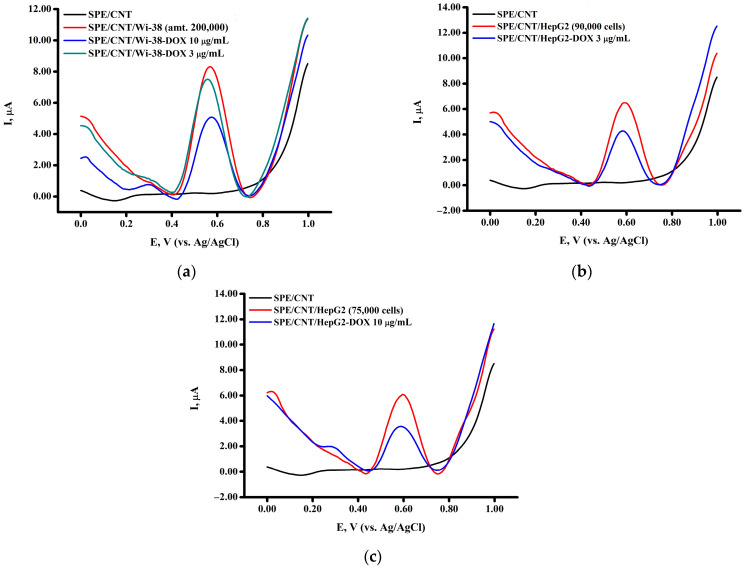
Square-wave voltammograms of (**a**) Wi-38 and (**b**,**c**) HepG2 cells treated with doxorubicin at concentrations of (**b**) 3 μg/mL and (**c**) 10 μg/mL. The study was performed with square-wave voltammetry in the potential range of 0 to +1.0 V. Peak intensities were recorded according to the number of cells per electrode (cells/electrode).

Evaluation of the effect of the cytotoxic drug doxorubicin on the cell lines Wi-38 and HepG2 showed a decrease in the intensity of the peak current depending on the concentrations 3 μg/mL and 10 μg/mL (Figure 4a–c). Our experiments confirmed the toxic effect of doxorubicin not only on the hepatocellular carcinoma cell line HepG2, but also on human fibroblast Wi-38 cells (Figure 4a). The influence of doxorubicin on the HepG2 cell line at the concentrations of 3 μg/mL and 10 μg/mL confirms the pronounced antitumor effect of this drug on human hepatocellular carcinoma cells (a decrease in peak current 1.56 and 2.76 times, respectively) (Figure 4b,c).

**Figure 5:**
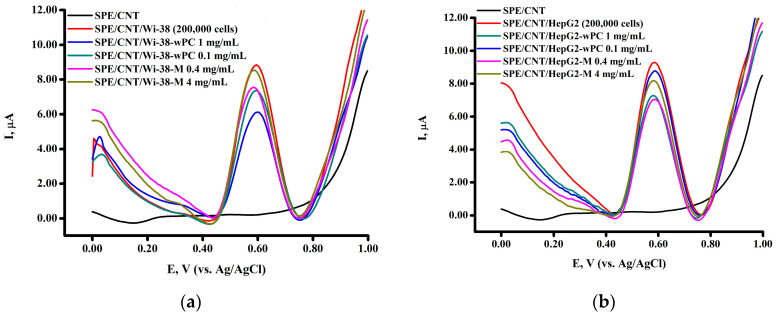
Square-wave voltammograms of (**a**) Wi-38 and (**b**) HepG2 cells treated with maltose monohydrate (M) (4 mg/mL and 0.4 mg/mL) and wPC at concentrations of 1 mg/mL and 0.1 mg/mL by PC. The study was performed with square-wave voltammetry in the potential range of 0 to +1.0 V.

**Figure 6:**
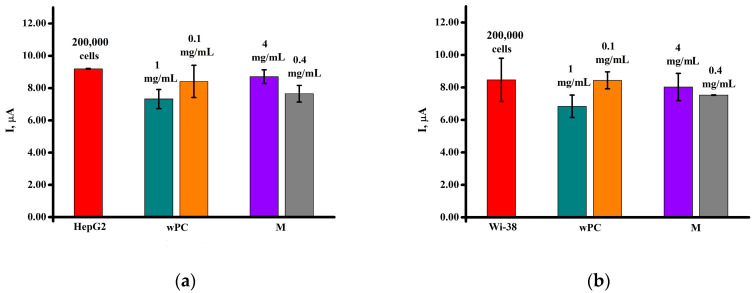
Histogram of the intensity of electrochemical oxidation of (**a**) Wi-38 and (**b**) HepG2 cells on electrode of untreated control and after incubation with maltose monohydrate (M) (concentrations of 4 mg/mL and 0.4 mg/mL) and wPC (concentration of 0.1 mg/mL and 1 mg/mL by PC).

The water-soluble form of phosphatidylcholine (wPC) at concentrations of 0.1 mg/mL and 1 mg/mL did not significantly influence the electrochemical signals of Wi-38 cells (Figure 5a and Figure 6a) in comparison with untreated control cells. It was also shown that the cryoprotector agent maltose also did not noticeably change the peak current intensity of the Wi-38 cell lines.

On the human hepatocellular carcinoma HepG2 cell line (Figure 5b and Figure 6b), a slight decrease in the peak current intensity was observed during incubation with wPC in comparison with untreated cells (control). A similar result was observed during incubation of cells with a cryoprotectant maltose. Based on the obtained results, the cytotoxicity of the studied samples was calculated using two methods, MTT and square-wave voltammetry (Table 1).

**Figure 7:**
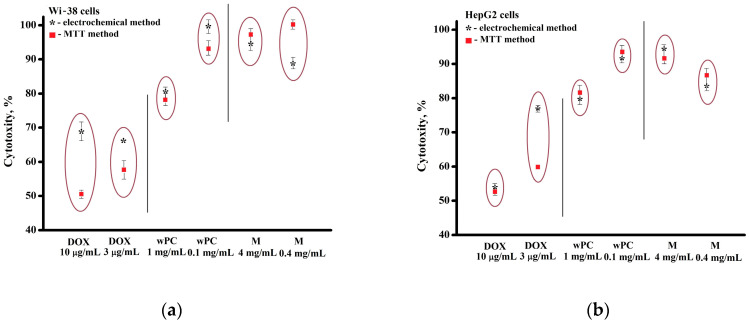
Comparative diagrams of cytotoxicity effects of the Wi-38 (**a**) and HepG2 **(b**) cells exposed to DOX, wPC, and maltose using the electrochemical method (*) and MTT assay (▪).

The results of the study of the cytotoxicity of wPC, maltose, and doxorubicin on the cell lines Wi-38 and HepG2 obtained by two methods, using the MTT test and electrochemical bioassay, based on registration of the cell-surface proteins showed their clear adequacy and similarity of the results obtained (Table 1, Figure 7).

## 4. Discussion

The problem of slowing down aging and the search for ways and opportunities to prolong lifespan are receiving great attention in the world. Pharmacological interventions that can increase life expectancy are of growing interest in this issue [55]. In this regard, great consideration in the world is being paid to the concept of geroprotectors aimed at biological aging to prevent many recurrent diseases [56].

The use of ultra-small plant phospholipids can reduce the level of non-HDL-C (non-high-density lipoprotein (HDL) cholesterol), triglycerides (TG), apolipoprotein B, total protein, and very low-density lipoprotein cholesterol, which allows them to be used in the treatment of combined hyperlipidemia [13,35]. In addition, the use of phospholipid-based drugs can reduce the risk of age-related diseases and play an important role in protecting from liver failure [57,58,59]. One of the criteria for increasing lifespan is the use of drugs that do not cause toxic effects or the selection of doses of drugs that do not have negative effects and unwanted side effects on the body [60,61].

Previously, it was shown that water-soluble ultra-small phospholipid nanoparticles-micelles are safe and effective for 12 weeks of oral administration for combined hyperlipidemia treatment. Phospholipid nanoparticles result in a significant decrease from baseline levels of non-HDL-C and TG [35]. It is known that in patients with ischemic heart disease (IHD) and acute coronary syndrome, the phosphatidylcholine/phosphatidylethanolamine (PC/PE) ratio in HDL is less than that in stable IHD patients. It is expected that the phospholipidation by phosphatidylcholine of these patients may stabilize the atherosclerotic plaques and prevent them from disruption followed by future thrombosis and myocardial infarction [60].

In several studies, a change in the molar ratio between PC and PE was established as a key determinant of liver health. Changes in the hepatic PC/PE ratio have been linked to the development of non-alcoholic fatty liver disease (NAFLD) as well as liver failure, impaired liver regeneration, and the severity of alcoholic fatty liver disease in humans [12]. Thus, oral long-term use of phospholipid nanoparticles may contribute to a protective effect against severe adverse cardiovascular complications and liver diseases and provide longevity for these patients.

Various classical analytical methods are used to analyze the toxicity of medicinal agents [61]. In recent years, more attention has been paid to alternative methods for studying drug toxicity, for example, using electrochemical biosensors. Cell-based biosensors are a hot topic in modern biosensor research. Thus, using a smartphone-based electrochemical sensor on HepG2 cells, the toxicity of heavy metal ions (Cd^2+^, Pb^2+^, Hg^2+^) was assessed [47]. Using various electrochemical sensors, toxicity analysis was carried out for tri(2-butylxyethyl) phosphate (TBEP), tributyl phosphate (TnBP), triphenyl phosphate (TPhP), tri(1,3-dichloro-2-propyl) phosphate (TDCIPP), tri(2-chloropropyl) phosphate (TCPP), tri(2-chloroethyl) phosphate (TCEP), acrylamide, and other compounds [23,61,62]. HepG2 liver cancer cells were analyzed by means of immunosensors based on the anti-CD133 antibody with the range of 1 × 10^5^ to 3 × 10^6^ cells/mL [63,64]. In our experiments, analysis of HepG2 and Wi-38 cell lines by square-wave voltammetry showed a linear dependence of the peak current intensity at the potential of E = +0.5 ÷ 0.6 V on cell concentration. The study of the water-soluble form of phosphatidylcholine (wPC) on HepG2 and Wi-38 cell lines at sufficiently high concentrations (1 mg/mL) showed an insignificant decrease in the peak current intensities of living cells after 24 h of incubation with the substance, which is consistent with the results obtained by the classical MTT assay. At the same time, the cryoprotector maltose as a component of wPC did not significantly affect cell survival. The electrochemical cyto-biosensor based on the direct reagent-free analysis of cells allows for an accurate assessment of the effect of drugs on cells’ viability.

## 5. Conclusions

Electrochemical mediator-free and reagent-free analysis of cell lines based on the registration of electrochemical profiles of membrane cell-surface proteins is an effective approach for studying both the specificity of cells and the toxic effects of drugs on cell-on-electrode systems. In this work, we presented a cyto-biosensor for two types of cells, Wi-38 and HepG2, in the concentration range from 1 × 10^5^ to 6 × 10^5^ cells/electrode. The studied water-soluble form of phosphatidylcholine, which previously demonstrated the effect of reducing the level of low-density cholesterol and hepatoprotective properties, also did not show a toxic effect on either normal human fibroblast cells (Wi-38) or human hepatocellular carcinoma cells (HepG2) at sufficiently high concentrations. In addition, a comparative analysis of the effect of the toxic agent doxorubicin and wPC on their toxic manifestations on cells was carried out. As a result, it was shown that doxorubicin (at 3–10 μg/mL concentration) causes a decrease in peak current intensity by almost 2 times compared with untreated cells. Treatment with wPC resulted in a decreased intensity of peak current of cell lines by 1.2 times at high concentrations (0.1–1 mg/mL). Thus, it is worth saying that electrochemical bioassay can be used both for monitoring cell growth and the effect of the environment on the growth rate and for assessing cell survival under the influence of various substances and in the search for new medications, in particular, cytostatics. The suggested non-invasive strategy for electrochemical analysis of mammalian cells could potentially be used for developing new therapeutics and comparative analysis of drugs under study.

The electrochemical detection of cells for drug toxicity analysis holds significant practical value and demonstrates considerable potential for rapid detection applications. Future perspectives of cell-based mediator-free and reagent-free biosensors may be described as investigation of the broad spectrum of eukaryotic cells for the analysis of specificity of registration and evaluation of toxic effects of exogenous substances. The limitation of our approach deals with the sensitivities of the electrodes for the registration of different types of cells. However, appropriate modification of the working surface of the electrode can improve this parameter of electrochemical analysis. Our results demonstrated two main points. The first one is that different drugs possess different effects, toxic or nontoxic, on cells, registered by means of the MTT test or electroanalytical method. The second one is that the water-soluble form of phosphatidylcholine (wPC) nanoparticles, recommended for atherosclerosis treatment and prevention of cardiovascular diseases [35], did not show a toxic effect on human fibroblast cells, Wi-38, or human hepatocellular carcinoma cells, HepG2, at sufficiently high concentrations (such as 0.1–1 mg/mL). Based on these data, wPC can be classified as a safe drug.

## Figures and Tables

**Table 1:** Cell viability of Wi-38 and HepG2 cell lines.

Sample	Cell Viability (Electrochemical Analysis), % ± SD	Cell Viability (MTT Assay), % ± SD
Wi-38 Cell Line		
DOX 10 µg/mL	68.9 ± 2.73	50.5 ± 1.24
DOX 3 µg/mL	65.41 ± 0.47	57.13 ± 3.23
wPC 1 mg/mL	80.6 ± 0.92	79.13 ± 2.60
wPC 0.1 mg/mL	99.6 ± 2.04	93.34 ± 2.20
Maltose 4 mg/mL	94.8 ± 2.48	97.0 ± 2.04
Maltose 0.4 mg/mL	88.9 ± 1.77	100 ± 0.97
HepG2 Cell Line		
DOX 10 µg/mL	53.2 ± 0.93	54.0 ± 1.04
DOX 3 µg/mL	77.7 ± 0.93	60.0 ± 0.53
wPC 1 mg/mL	79.7 ± 0.36	81.0 ± 2.8
wPC 0.1 mg/mL	91.5 ± 1.00	93.0 ± 2.4
Maltose 4 mg/mL	94.8 ± 0.18	92.0 ± 1.95
Maltose 0.4 mg/mL	83.2 ± 0.27	86.0 ± 2.7

## Data Availability

The original contributions presented in this study are included in the article. Further inquiries can be directed to the corresponding author.
